# *In vitro* antimicrobial activity and HPTLC analysis of hydroalcoholic seed extract of *Nymphaea nouchali* Burm. f.

**DOI:** 10.1186/1472-6882-14-361

**Published:** 2014-09-26

**Authors:** Mabel Parimala, Francis Gricilda Shoba

**Affiliations:** P.G. & Research Department of Zoology, Voorhees College, Vellore, Tamilnadu 632001 India

**Keywords:** *Nymphaea stellata*, Phytochemicals, Antibacterial, Catechin, MIC, Agar disc diffusion, Herbal drug

## Abstract

**Background:**

In the Indian ayurvedic system of medicine, *Nymphaea nouchali* is used for the treatment of diabetes, cutaneous diseases, inflammation, liver disorders, urinary disorders, menorrhagia, blenorrhagia, menstruation problem, as an aphrodisiac, and as a bitter tonic. However, despite its traditional usage as an antimicrobial agent, there is no information regarding its effectiveness in infections caused by pathogenic microbes. Hence, we evaluated 70% ethanol extract of the seeds of *N. nouchali* for its antimicrobial activity.

**Methods:**

The antimicrobial activity of the extract at five different concentrations was tested against few common human pathogenic microorganisms by agar disc diffusion assay. The Minimum Inhibitory Concentration of the extract was determined by the modified resazurin method. Streptomycin (10 μg/ml) and amphotericin B (10 μg/ml) were used as standards for antibacterial and antifungal study respectively. Few phenolic compounds were identified and quantified by standard HPTLC technique.

**Results:**

The zone of inhibition was extremely great for *P. aeruginosa* (25 mm), *S. aureus* (20 mm) and *C. albicans* (19 mm). MIC value was the least at 0.03 mg/ml for bacteria: *K. pneumoniae, S. dysenteriae* and *E. coli* and 0.31 mg/ml for fungi: *C. albicans* and *T. mentagrophytes*. Moreover, through HPTLC analysis few phenolic compounds were quantified, among which catechin content was found to be the highest (3.06%), followed by gallic acid (0.27%) and quercetin (0.04%).

**Conclusions:**

The results therefore clearly indicates that the crude extract from *N. nouchali* seeds could be used as a potential source of natural antimicrobial agent owing to the presence of the phytoconstituent catechin in abundance along with other active compounds and supports the traditional use of the plant in the treatment of infections.

## Background

Infectious diseases are disorders caused by bacteria, fungi, virus or parasites [1]. According to the WHO, infectious diseases are a significant cause of morbidity and mortality worldwide, accounting for 50% of all deaths in tropical countries. During the last decade, several reports indicated that bacterial strains are resistant against almost all clinically available antibiotics [2, 3]. Certainly the wide use and misuse of antibiotics have led to a situation of spreading resistant bacteria. The first line antimicrobials were both effective and affordable; however with the onset of resistance, newer treatments are proving too costly. They are not without side effects. Therefore the search for new antimicrobial agents is becoming a hot topic since fatal opportunistic infections are registered every year.

Although many have been treated by conventional pharmaceutical approaches, there is a growing interest in the use of natural products by general public [4]. Medicinal plants are found to be a better choice with wide range of bioactive compounds [5]. They are perceived to be non-toxic, safe, effective and sometimes the only source of health care available to the poor [6]. That is why nearly 80% of the people are dependent, wholly or partially on plant-based drugs [7]. Indeed, medicinal plants with antimicrobial properties are being increasingly reported from different parts of the world. Natural products especially from higher plants open up a new source of antimicrobial agents with possible novel mechanisms of action [8]. The phytoconstituents of such plants are responsible for their pharmacological activity. Secondary metabolites of plant origin appear to be one of the alternatives for the control of antibiotic-resistant human pathogens. They become the base for the development of new drugs. The wide arrays of active compounds affect the behaviour of microorganisms in such a way that they are toxic to the pathogens [9]. However due to their antibacterial, antifungal and antiviral activity, phenolic compounds of plant origin have gained interest in the recent years [10].

*Nymphaea nouchali* (synonym: *Nymphaea stellata*), is a perennial aquatic herb belonging to the family Nymphaeaceae. It is commonly known as the white water lily. It is an important and well-known medicinal plant, widely used in Ayurveda and Siddha system of medicines for the treatment of diabetes, inflammation, liver disorders, urinary disorders and as a bitter tonic. The fruit of this plant is globose containing round, flask-shaped seeds. The seeds are used as stomachic and restorative. The seeds are also prescribed as diet for diabetes in the ayurvedic system of medicine [11]. Earlier studies have reported the seeds to possess significant antioxidant [12], antidiabetic [13] and antihepatotoxic activity [14]. Glycosides, phenols, tannins, flavonoids, saponins and alkaloids are also reported in the seeds [12]. Recently, nymphasterol, a new steroid has been isolated and identified from the seeds [15].

Our study was focused on the evaluation of hydroalcoholic extract of *N. nouchali* seeds against few pathogenic microbes. Standardization of thin layer chromatograms for the identification and quantification of certain phenolic compounds has been done. This is the first report on the antimicrobial activity of *N. nouchali* seeds with subsequent HPTLC analysis.

## Methods

### Chemicals and reagents

Culture media for microbial studies were obtained from Himedia Laboratories Pvt. Ltd., Mumbai, India. Pure compounds quercetin, gallic acid and catechin for HPTLC studies were procured from Sigma Chemicals, Bangalore, India. Silica gel GF_254_ TLC plates were purchased from Merck (Darmstadt, Germany). Other solvents and chemicals used were of analytical grade.

### Plant material

Seed samples were collected from the plant *N. nouchali* at a pond in Kanyakumari District, India. The plant was identified by Prof. Dr. Jayaraman, Plant Anatomy Research Centre (PARC), Chennai and a voucher specimen was deposited at the herbarium of PARC for future reference [PARC/2012/1248]. The samples were collected into plastic zip-lock bags with appropriate labeling and stored until it was taken to the laboratory. The seeds were then washed with distilled water and open air-dried away from sunlight.

### Preparation of plant extract

Extraction was performed by hot percolation method using soxhlet apparatus. About 250 g of the coarsely powdered *N. nouchali* seeds was extracted in 500 ml of 70% ethanol by continuous hot extraction method at 50°C. The extract with the solvent was decanted from the soxhlet apparatus and the filtrate was evaporated for the total elimination of solvent using a Rota flash vacuum evaporator at 50°C. The concentrated liquid extract obtained was then transferred to a china dish and kept in a water bath to dry. The residual extract (NHS) was transferred and stored in an air tight container.

### Microorganisms

A total of ten bacterial cultures *(Bacillus cereus, Brucella, Enterococcus faecalis, Escherichia coli, Klebsiella pneumoniae, Pseudomonas aeruginosa, Salmonella typhi, Shigella dysenteriae, Staphylococcus aureus, Vibrio cholerae*) and five fungal cultures (*Aspergillus niger, Candida albicans, Curvularia, Penicillium, Trichophyton mentagrophytes*) were used in this study. All the cultures were obtained from Royal Bioresearch Centre, Velachery, Chennai. The cultures were stored on nutrient agar slants at 4°C and were sub-cultured on nutrient agar medium before antimicrobial testing.

### Inoculum preparation

The starter cultures, in tubes with 2 ml of nutrient broth were inoculated with the microorganisms for bioassay and incubated for 24 h time period at 37°C.

### Determination of antimicrobial activity

Antimicrobial activity was tested using disc diffusion assay [16]. Mueller Hinton Agar and Potato Dextrose Broth were used for bacterial and fungal susceptibility test respectively. The nutrient mediums were transferred into one-fourth volume of the plate. After solidification, the inoculums were spread on the solid plates with sterile swab moistened with the microbial suspension. Different concentrations (1000, 500, 250, 125, 62.5 μg/ml) of NHS extract in 1% v/v DMSO were prepared as individual stocks in sterile vials. Whatman filter No.1 paper discs were soaked with the extracts and air-dried thoroughly before the assay. The plates were incubated for 24 h at 37°C. The inhibition of bacterial and fungal growth was determined by measuring the diameter of the clear zone around each disc. The sterile discs soaked with 1% DMSO served as negative control. Standard antibiotics: streptomycin, 10 μg/ml and amphotericin B, 10 μg/ml were used as standards for antibacterial and antifungal test respectively. Average of triplet readings for each microorganism was recorded.

### Determination of minimum inhibitory concentration

The minimum inhibitory concentration (MIC) was determined by the modified resazurin method [17]. Test was carried out in a 96 well plate under aseptic conditions. A volume of 100 μl of test material (a stock concentration of 10 μg/ml for standards and 10 mg/ml for NHS extract in 1% DMSO) was pipetted into the first row of the plate. To all other wells 50 μl of nutrient broth was added and serial dilutions were performed. To this resazurin indicator solution was added. 30 μl of 3.3X strength isosensitised broth was added to each well to ensure that the final volume was single strength of the nutrient broth. Finally, 10 μl of bacterial suspension was added to each well to achieve a concentration of 5 × 10^5^ cfu/ml (for bacterial isolates) and/or 10 μl of fungal suspension was added to each well to achieve a concentration of 10^5^ spores/ml (for fungal isolates). Each plate had a set of controls. The plates were prepared in triplicate, and placed in an incubator set at 37°C for 24 h. The colour change was then assessed visually. Any colour change from purple to pink was recorded as positive. The lowest concentration at which colour change occurred was taken as the MIC value.

### Thin layer chromatography study

Based on the preliminary qualitative phytochemical screening, TLC studies were performed with known standards. A Camag TLC system equipped with Camag Linomat V, an automated TLC sample spotter, Camag glass with trough chamber (20 × 10 cm) was used for the analysis. The NHS extract was separated in suitable mobile phase along with standards.

### Identification and quantification of quercetin

A stock solution of quercetin (100 μg/ml) was prepared in methanol. Working solutions were prepared by appropriate dilution of the stock solution with the same solvent. Quantification was performed by external standard method using pure quercetin as standard. Sample solution was applied on the TLC plate and developed with mobile phase chloroform: ethyl acetate: formic acid: methanol (2.5:2:0.4:0.2, v/v/v/v). Densitometric scanning was performed at 412 nm. Peak areas were recorded and the amount of quercetin was calculated using the calibration curve.

### Identification and quantification of gallic acid

A stock solution of gallic acid (100 μg/ml) was prepared in methanol. Working solutions were prepared by appropriate dilution of the stock solution with the same solvent. Quantification was performed by external standard method using pure gallic acid as standard. Sample solution was applied on the TLC plate and developed with mobile phase chloroform: ethyl acetate: formic acid (2.5:2:0.8, v/v/v). Densitometric scanning was performed at 280 nm. Peak areas were recorded and the amount of gallic acid was calculated using the calibration curve.

### Identification and quantification of catechin

A stock solution of catechin (1 mg/ml) was prepared in methanol. Working solutions were prepared by appropriate dilution of the stock solution with the same solvent. Quantification was performed by external standard method using pure catechin as standard. Sample solution was applied on the TLC plate and developed with mobile phase chloroform: ethyl acetate: formic acid: methanol (2.5:2:0.8:0.2, v/v/v). Densitometric scanning was performed at 280 nm. Peak areas were recorded and the amount of catechin was calculated using the calibration curve.

## Results

### Antibacterial activity

**Figure 1:**
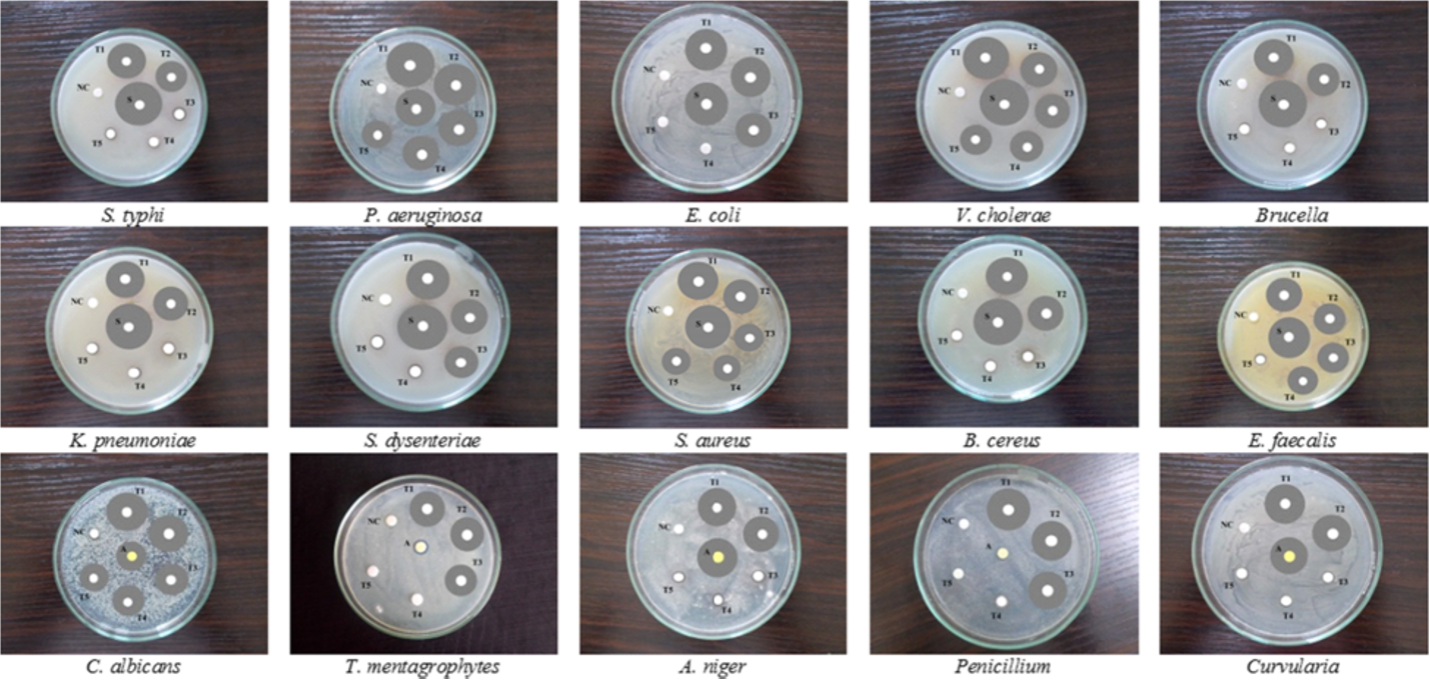
**Antimicrobial activity of** ***Nymphaea nouchali*** **seed extract using disc diffusion assay.** Culture plates showing zones of inhibition. T1: 1000 μg/ml NHS, T2: 500 μg/ml NHS, T3: 250 μg/ml NHS, T4: 125 μg/ml NHS, T5: 62.5 μg/ml NHS, NC: Negative control 1% DMSO, S: Streptomycin, A: Amphotericin B.

The antibacterial activity of NHS extract varied depending on the various concentrations used and the tested microorganisms (Figure 1). The zones of inhibition ranged between 8 mm and 25 mm diameter. Almost all the microorganisms were susceptible to NHS extract, though in different concentrations. The present study revealed that the crude extract possessed significant inhibitory activity against *P. aeruginosa, S. aureus* and *V. cholerae* even at low concentration of 62.5 μg/ml. The growth of *E. faecalis* was inhibited at 125 μg/ml of extract. The extract also effectively inhibited *E. coli* and *S. dysentriae* at 250 μg/ml concentration. The rest of the tested microorganisms started forming zones of inhibition at high concentrations of 500 μg/ml. However at 1000 μg/ml, the highest antibacterial activity was recorded against *P. aeruginosa* (25 mm), followed by *S. aureus* (20 mm), *E. coli* (15 mm), *B. cereus* (14 mm) and *S. dysenteriae* (14 mm) and the least activity was recorded against *E. faecalis* (11 mm). Surprisingly, the NHS extract at 500 μg/ml concentration, effectively suppressed *P. aeruginosa* (20 mm) which was greater than the standard antibiotic streptomycin (17 mm) used as reference (Table 1).

**Table 1:** **Antibacterial activity of** ***Nymphaea nouchali*** **seed extract in different concentrations**

Microorganisms	Zone of inhibition (mm)						
	1000 μg/ml	500 μg/ml	250 μg/ml	125 μg/ml	62.5 μg/ml	DMSO 1% v/v	Streptomycin 10 μg/ml
Gram negative bacteria							
*S. typhi*	12	9	-	-	-	-	19
*P. aeruginosa*	25	20	17	15	11	-	17
*E. coli*	15	14	11	-	-	-	16
*V. cholerae*	13	10	9	9	8	-	20
*Brucella*	13	8	-	-	-	-	20
*K. pneumoniae*	13	11	-	-	-	-	19
*S. dysenteriae*	14	9	8	-	-	-	20
Gram positive bacteria							
*S. aureus*	20	18	15	12	11	-	23
*B. cereus*	14	9	-	-	-	-	23
*E. faecalis*	11	9	8	8	-	-	19

### Antifungal activity

The NHS extract also showed significant fungal growth inhibition against all the tested fungi comparable to the standard amphotericin B (Table 2). Among the five tested fungi, the best antifungal activity was obtained with the extract against *C. albicans* showing 19 mm diameter inhibitory zone. Moreover at 62.5 μg/ml concentration of the drug, notable inhibition similar to the standard used was observed. Interestingly, *T. mentagrophytes* and *Penicillium* were susceptible to NHS extract at 250 μg/ml, while amphotericin B did not exhibit fungicidal property against these two species at all (Figure 1).

**Table 2:** **Antifungal activity of** ***Nymphaea nouchali*** **seed extract in different concentrations**

Microorganisms	Zone of inhibition (mm)						
	1000 μg/ml	500 μg/ml	250 μg/ml	125 μg/ml	62.5 μg/ml	DMSO (1% v/v)	Amphotericin B (10 μg/ml)
*C. albicans*	19	18	15	13	11	-	11
*T. mentagrophytes*	9	8	7	-	-	-	-
*A. niger*	10	9	-	-	-	-	10
*Penicillium*	10	9	8	-	-	-	-
*Curvularia*	11	10	-	-	-	-	10

### Minimum inhibitory concentration (MIC)

**Figure 2:**
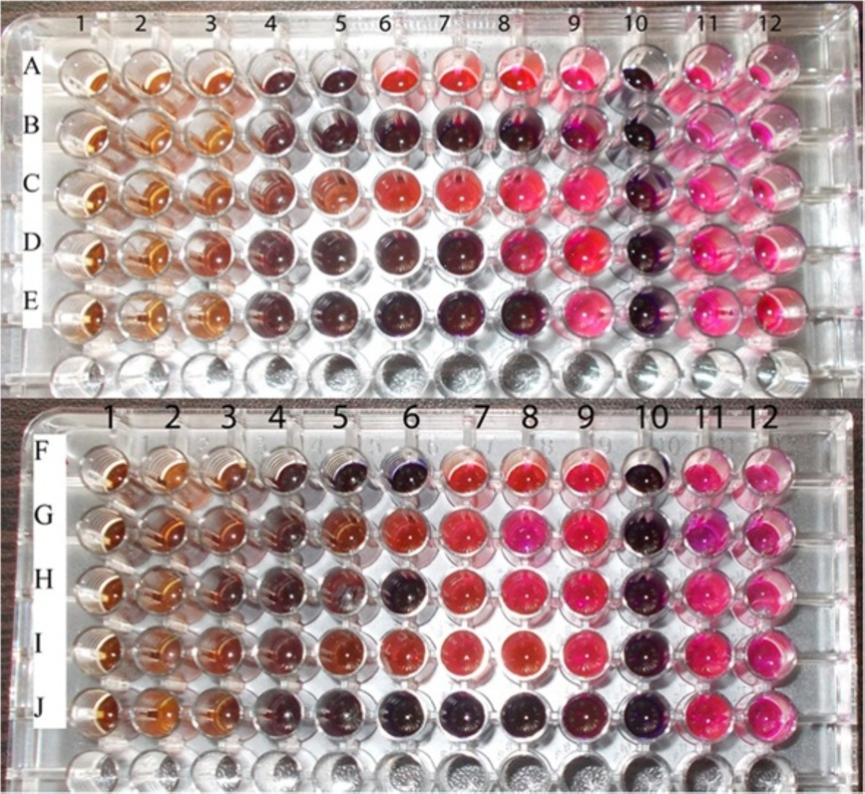
**Plates showing MIC of** ***Nymphaea nouchali*** **seed extract against the tested bacteria.** **A**: *B. cereus*, **B**: *K. pneumoniae*, **C**: *S. aureus*, **D**: *E. faecalis*, **E**: *S. dysenteriae*, **F**: *V. cholerae*, **G**: *P. aeruginosa*, **H**: *Brucella*, **I:** *S. typhi*, **J**: *E. coli*, 1 – 9: 10, 5, 2.5, 1.25, 0.62, 0.31, 0.15, 0.07, 0.03 mg/ml NHS, 10: streptomycin, 11: Control containing DMSO, 12: Culture alone.

**Figure 3:**
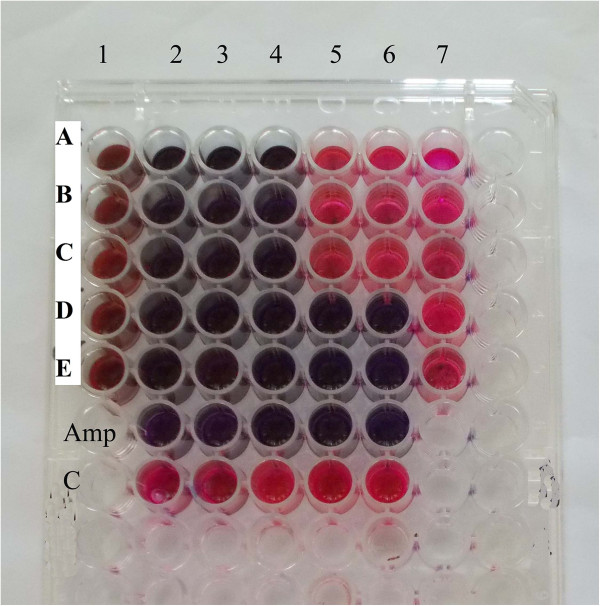
**Plate showing MIC of** ***Nymphaea nouchali*** **seed extract against the tested fungi.** **A**: *Curvularia*, **B**: *A. niger*, **C**: *Penicillium*, **D**: *T. mentagrophytes*, **E**: *C. albicans*, 1: NHS without culture, 2–7: 10, 5, 2.5, 1.25, 0.62, 0.31 mg/ml NHS, Amp: Amphotericin B, C: Control containing DMSO.

Among the ten tested bacterial strains, the extract had the lowest MIC (0.03 mg/ml) for *K. pneumoniae, S. dysenteriae* and *E. coli* and the extract had the highest MIC (0.62 mg/ml) for *S. aureus* (Table 3). The MIC values for the remaining microorganisms ranged between 0.07 mg/ml and 0.31 mg/ml (Figure 2). On the other hand, with respect to fungi (Figure 3), the MIC was the least for *C. albicans* and *T. mentagrophytes* (0.31 mg/ml) (Table 4).

**Table 3:** **Minimum Inhibitory Concentration of** ***Nymphaea nouchali*** **seed extract against the tested bacteria**

Bacteria	MIC (mg/ml)
*B. cereus*	0.31
*K. pneumoniae*	0.03
*S. aureus*	0.62
*E. faecalis*	0.07
*S. dysenteriae*	0.03
*V. cholerae*	0.15
*P. aeruginosa*	0.31
*Brucella*	0.15
*S. typhi*	0.31
*E. coli*	0.03

**Table 4:** **Minimum Inhibitory Concentration of** ***Nymphaea nouchali*** **seed extract against fungi**

Fungi	MIC (mg/ml)
*Curvularia*	1.25
*A. niger*	1.25
*Penicillium*	1.25
*T. mentagrophytes*	0.31
*C. albicans*	0.31

### HPTLC analysis

**Figure 4:**
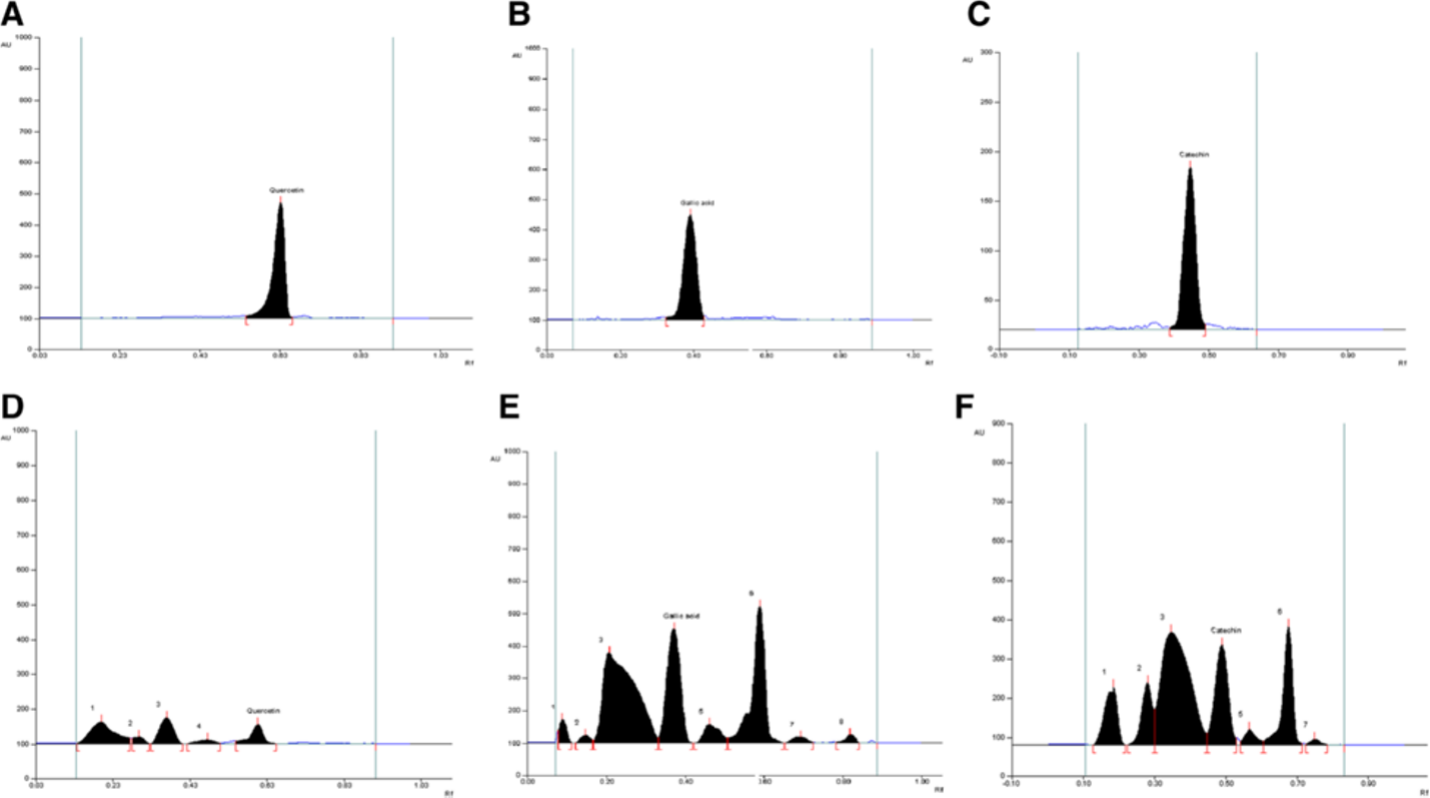
**HPTLC fingerprint profile. A:** Standard quercetin, **B:** Standard gallic acid, **C:** Standard catechin, **D:** Quercetin in NHS extract, **E:** Gallic acid in NHS extract, **F:** Catechin in NHS extract.

HPTLC analysis was performed for three phenolic phytocompounds namely: quercetin, gallic acid and catechin. The amount of quercetin in NHS extract was estimated to be 0.04%. Similarly, the obtained value of gallic acid was 0.27% of the crude drug. Catechin content in the extract was also evaluated and found to be 3.06% of NHS drug (Figure 4).

## Discussion

Antimicrobial activity can be regarded as a very important study, particularly at this specific period of human history due to bacterial resistance that is constantly imposing new scientific challenges. In spite of great development in drug therapy, infectious diseases remain as the most common reasons for mortality in many developing countries. Resistance to antimicrobial agents has become an increasingly important and pressing global problem. To counter the emergence of resistant microorganisms, considerable resources have been invested in the search for new antimicrobials.

It is well known that plants synthesize a diverse array of secondary metabolites, which are involved in defense mechanisms, and in the last few years it is recognized that some of these molecules have health beneficial effects including antimicrobial properties [18]. According to the WHO, plants are a source of compounds that have the ability to combat disease. *Nymphaea nouchali* is a well-known medicinal plant in the Indian traditional system of medicine. Due to their various biological activities, the plant and its compounds are continuously under investigation.

Hence the present study was focused in evaluating the antimicrobial property of the hydroalcoholic seed extract of *N. nouchali*. The NHS extract showed notable antibacterial activity against *S. typhi, P. aeruginosa, E .coli* and *V. cholerae.* It also showed remarkable activity against *S. aureus* and *B. cereus*. Thus it is clear that the NHS extract is effective in controlling both gram-negative and gram-positive bacteria. This evidence opens possibilities to the fact that the extract contains compounds that may act by synergism or additive effect which in turn is responsible for its pharmacological activity [19].

Among the phytocompounds, polyphenols or phenolic compounds are a group of secondary metabolites that are adequately found in medicinal plants with more than 8000 identified compounds [20], and have been tested in clinical and experimental studies as antimicrobials [21]. Quality control which is an important challenge in present scenario can be addressed with reliable and sensitive quantization of important biological active metabolite in the sample [22]. High Performance Thin Layer Chromatography is one of the modern sophisticated techniques than can be used for evaluating the potency, authenticity, quality and purity of crude drugs [23]. HPTLC offers better resolution, and estimation of active constituents can be done with reasonable accuracy in a shorter time [24]. Polyphenols found in medicinal plants have been extensively investigated against a wide range of microorganisms, and among them tannins and flavanols received more attention due to its broad spectrum and the fact that most of them are able to suppress microbial virulence factors [25]. So the present study also demonstrates the quantization of few phenolic compounds like quercetin (Q), gallic acid (GA) and catechin (CA).

Flavonoids like quercetin and catechin are becoming the subject of medical research [26]. Flavonoids interfere with specific intracellular or surface enzymes and many bacterial virulence factors such as toxins, enzymes and signal receptors [27]. *Xanthomonas phaseoli* showed a similar antimicrobial activity confirming that flavonoids may have been responsible for its activity [28]. CA content in NHS extract was the highest among the tested compounds. CAs are a group of flavonoids that have been extensively researched due to their occurrence in green teas. CAs from *Camellia sinensis* showed profound antibacterial activity against *Shigella*, *Vibrio* and *S. mutans* [29]. The CAs in NHS extract might have inactivated cholera toxin in *V. cholerae* due to complexing mechanism similar to that which has been reported earlier [30]. CAs appear to have greater activity against gram-positive than gram-negative bacteria due to the presence of lipopolysaccharide acting as a barrier [31]. However, recently it has been established that in gram-negative bacteria, cell surface structures lacking O antigen and core polysaccharide have increased the sensitivity of CAs [32]. Black tea polyphenols are also known to have antibacterial effects against *B. cereus*, *Staphylococci* and methicillin-resistant *S. aureus* [33].

Increased level of CA and epicatechin in the regenerated bark and leaves of *Saraca asoca* showed importance of these metabolites in prevention of infection [22]. 3-o-octanoyl CA have shown to cause a reduction of 1000-fold or more in viable counts of MRSA-YK, *S. aureus* NCTC 6571 and EMRSA-16 due to the formation of pseudomulticellular aggregates both in antibiotic sensitive and antibiotic resistance strains of *S. aureus* [34]. Sub-inhibitory concentrations of tea CAs have also suppressed the opportunistic pathogen *S. aureus* to β-lactam antibiotics and relatively low concentration of epicatechin have sensitized MRSA clinical isolates to levels of oxacillin by affecting both virulence and antibiotic resistance by perturbing the function of key processes associated with the bacterial cytoplasmic membranes [35].

Antimicrobial effects of CAs were also observed against *Staphylococcus* strains, which cause suppuration-related inflammation and bedsores, and against *Candida*, which causes oral candidiasis, suggesting that NHS extract may aid in the prevention of oral candidiasis and suppuration [36]. There are several reports about the potential antifungal activity of polyphenols for treating oral candidiasis. Gel-entrapped catechins (GEC) inhibited the growth of *Candida* strains suggesting that hydrogen peroxide may be involved in the antimicrobial activity of CA [37]. Black tea extract containing CA completely inhibited the growth of *T. mentagrophytes* [38]. Surprisingly, the *C. albicans* was unaffected with heat treatment of tea CAs [39].

GA is reported to be highly antimicrobial against gram-negative pathogens. *Salmonella* tested against GA showed to have varying degrees of antimicrobial activity. Studies have reported *B. cereus* isolates sensitive to GA [40]. On examination of the activity of three extracts from the fruiting bodies of the tree *Terminalia chebula* RETS against methicillin-sensitive and MRSA, GA derivatives were more effective against both types of *S. aureus* [41]. *E. coli* was observed to have a very similar antimicrobial activity against GA with MIC of 15 ppm after 60 h of incubation [42]. GA had antimicrobial activity against the bacteria tested, with MIC of 500 μg/ml for *P. aeruginosa*; 1500 μg/ml for *E. coli* and 1750 μg/ml for *S. aureus*. GA is believed to cause irreversible changes in membrane properties through hydrophobicity changes, decrease of negative surface charge and occurrence of local rupture or pore formation in the cell membranes with consequent leakage of essential intracellular constituents [18]. Therefore, GA in NHS could have partly contributed to its action against gram-negative microorganisms in the study.

Hence, the antimicrobial activity may be due to the presence of catechin and gallic acid in the extract which have been previously reported for their antimicrobial property. It is therefore conceivable that this extract could be used against infections caused by the microbes against which it has shown pronounced effects. The results showed a good correlation between the reported uses of *N. nouchali* in traditional medicine against infectious diseases. This study may not be adequate to suggest potential antibiotic agent considering the MIC values and the zones of inhibition. However, this approach could be considered as preliminary step to find out promising lead molecules and the possible mechanism of action by which it inhibits microorganisms.

## Conclusions

The demonstration of activity against both gram-negative and gram-positive bacteria, and fungi is an indication that *Nymphaea nouchali* seeds can be a source of bioactive substances. The study also reports HPTLC analysis of quercetin, gallic acid and catechin from *N. nouchali* seed extract. The extract containing catechin and gallic acid must have contributed to its antimicrobial activity. The overall study, thus, emphasizes the potency of *N. nouchali* as a green and sustainable source of new broad spectrum of antimicrobial products. Because of its interesting biological activity, specialists can focus their increasing interest towards this plant. However, further research is required to determine the compound that has contributed to the antimicrobial activity of the extract and its exact mode of action.
